# Reinventing the public square and early educational settings through culturally informed, community co-design: Playful Learning Landscapes

**DOI:** 10.3389/fpsyg.2022.933320

**Published:** 2022-12-07

**Authors:** Annelise Pesch, Karlena D. Ochoa, Katelyn K. Fletcher, Vanessa N. Bermudez, Rachael D. Todaro, Julie Salazar, Hailey M. Gibbs, June Ahn, Andres S. Bustamante, Kathy Hirsh-Pasek

**Affiliations:** ^1^Department of Psychology and Neuroscience, Temple University, Philadelphia, PA, United States; ^2^School of Education, University of California, Irvine, Irvine, CA, United States; ^3^Center for American Progress, Washington, DC, United States; ^4^Brookings Institution, Washington, DC, United States

**Keywords:** guided play, playful learning, informal learning, urban design, education, research–practice partnership, community-based research, human-centered design

## Abstract

What if the environment could be transformed in culturally-responsive and inclusive ways to foster high-quality interactions and spark conversations that drive learning? In this article, we describe a new initiative accomplishing this, called Playful Learning Landscapes (PLL). PLL is an evidence-based initiative that blends findings from the science of learning with community-based participatory research to transform physical public spaces and educational settings into playful learning hubs. Here, we describe our model for conducting this research, which is mindful of three key components: community input, *how* children learn best, and *what* children need to learn to be successful in the 21st century economy. We describe how this model was implemented in two PLL case studies: one in a predominantly Latine community and the second in early childhood education classrooms. Furthermore, we describe how research employing our model can be rigorously and reliably evaluated using observational and methodological tools that respond to diverse cultural settings and learning outcomes. For example, our work evaluates how PLL impacts adult–child interaction quality and language use, attitudes about play and learning, and community civic engagement. Taken together, this article highlights new ways to involve community voices in developmental and educational research and provides a model of how science can be translated into practice and evaluated in culturally responsive ways. This synthesis of our process and evaluation can be used by researchers, policymakers, and educators to reimagine early educational experiences with an eye toward the built environment that children inhabit in everyday life, creating opportunities that foster lifelong learning.

## Introduction

Children from under-resourced homes often have less access to informal learning experiences (DeWitt and Archer, 2017; Takeuchi et al., 2019) and high-quality language interactions (Hirsh-Pasek et al., 2015; Pace et al., 2017; Golinkoff et al., 2019). Yet, these experiences support the development of skills foundational to science, technology, engineering, and mathematics (STEM) learning and are important predictors of later academic achievement (Ramani and Siegler, 2014; Verdine et al., 2014b; Bustamante et al., 2018; Hanner et al., 2019). Recent scholarship highlights that STEM learning can happen anywhere, in the activities present in a child’s daily routine (Ahn et al., 2018; Bustamante et al., 2019). For example, children often spend time with caregivers at the grocery store, walking to school, at the laundromat, waiting for the bus, or in neighborhood parks. These places, among others, often hold significance as spaces families frequently visit, where they gather with others and build community. A reinvention of the public square – community spaces where people gather – that draws on the science of learning offers an innovative way to transform everyday spaces into accessible and inclusive hubs that enable the experiences supportive of children’s learning (Hassinger-Das et al., 2021; Hadani et al., 2021).

**FIGURE 1:**
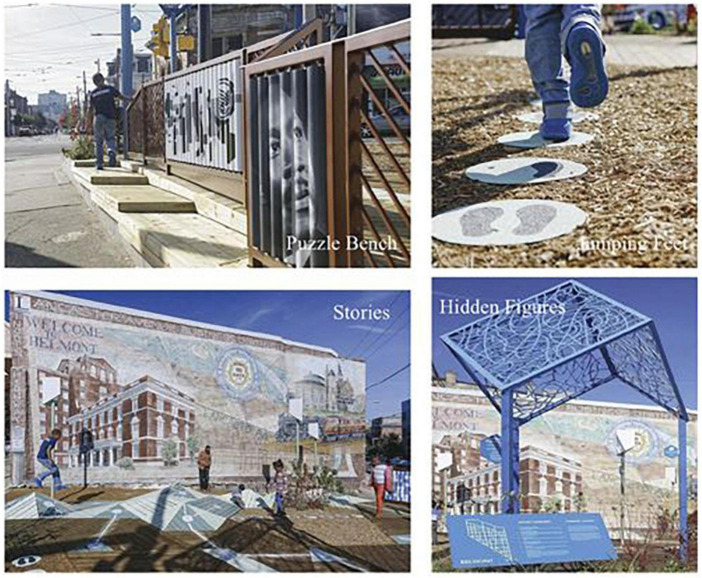
Urban Thinkscape installations from Hassinger-Das et al. (2020).

Playful Learning Landscapes (PLL) is a new initiative that takes what researchers know about child development and embeds these insights into everyday spaces. For example, executive functioning (EF) skills are foundational for later academic achievement (Diamond, 2012; Morgan et al., 2019) and have been the target of intervention work seeking to close educational opportunity gaps (Waters et al., 2021). What if designed environments encouraged families and children to exercise their EF skills in spaces where they already spend time together? PLL offers a way to do this with a game called Jumping Feet (see Figure 1). Jumping Feet is a version of the familiar sidewalk game, Hopscotch, with a new design and simple prompts that encourage children to jump onto tiles with one foot where they see two and two feet where they see one, activating the EF skills of inhibition and cognitive flexibility (Hassinger-Das et al., 2020). Jumping Feet was used at the first established PLL called Urban Thinkscape, a bus stop in an under-resourced community in Philadelphia, Pennsylvania, where children and caregivers take advantage of time spent waiting for the bus by playing games that foster important developmental skills, such as EF. In addition to Jumping Feet, Urban Thinkscape includes a puzzle wall activity that supports spatial awareness, a game called Stories that facilitates narrative skills and literacy, and finally, a game called Hidden Figures that encourages children and caregivers to identify shapes in shadows. Each of these activities promotes the development of skills (e.g., spatial skills, narrative development, and making observations) crucial to later academic achievement (Dickinson et al., 2010; Verdine et al., 2014a; Bower et al., 2020).

In addition to cultivating important academic and social skills, Urban Thinkscape was designed to reflect and uphold the surrounding community’s cultural values and history. A research–practice partnership (RPP) between university researchers and local grassroots organizations led to a series of town hall-style meetings and focus group sessions where community members voiced their goals and priorities for the space. The community identified and helped secure the lot where Urban Thinkscape stands, which holds important historical significance because of its proximity to where Martin Luther King, Jr. delivered a key speech in 1965. Community feedback further led to the inclusion of Martin Luther King, Jr. as a central image featured on the puzzle wall. After the site was secured and the designs approved, hundreds of community members came together to help build the activities at Urban Thinkscape. This process from start to finish offers a powerful example of the importance of community involvement in RPPs to ensure local representation and ownership of community spaces.

Evaluation of Urban Thinkscape revealed that changing an everyday space where families gather changed behavior in ways that fostered higher quality language and interaction between children and their caregivers. Trained data ambassadors, who were members of the local community, observed the amount of back-and-forth conversation between caregivers and children, the type of language used, and interaction quality. After Urban Thinkscape was installed, observers reported an increase in the amount of conversation between caregivers and children, more frequent use of spatial, numerical, and literacy language, and overall higher-quality interactions (Hassinger-Das et al., 2020). These results highlight that PLL impacts how families engage with each other in their local community spaces, and that it can do so with community involvement in culturally responsive and inclusive ways (Todaro et al., 2022).

Urban Thinkscape demonstrated a new way to conduct research by changing physical public spaces in concert with community values and then evaluating how this environmental change impacts caregiver–child interactions and child outcomes known to bolster later cognitive and academic achievement. In this article, we describe our model and process for implementing PLL, which highlights new ways to involve community voices in developmental and educational research. Specifically, we describe how this model was implemented in two PLL case studies, offering examples of extension and refinement of the work to new cultural settings and in the service of different learning outcomes. Finally, we discuss how our model for creating PLLs can be rigorously and reliably evaluated.

## The model

Our process for creating PLLs adheres to a new model for conducting developmental and educational research which takes three factors into account: community input and values, the science of *how* children learn, and the science of *what* children need to learn to thrive in the 21st century. In short, this model relies on an equation that melds community and science in ways that foster rich experiential learning and is culturally responsive and inclusive.

### Community input

PLL is not the first to discover that everyday spaces can influence human behavior in positive ways. It is the first to build designs informed both by community input and by the science of *how* and *what* children learn. Our method of ensuring community ownership and input in PLLs takes advantage of community-based participatory research (CBPR), an approach to research that involves active and ongoing community partnership (Hacker, 2013; Collins et al., 2018). Community-based participatory research (CBPR) lies on a continuum from projects that are investigator-initiated with partners playing a smaller role to projects where the procedures and methods are co-designed. Most psychological experiments do the former. PLL is an example of the latter in which co-design ensures that community members have an equal voice in informing research protocols, in decision-making, and in the final product of the research (Belgrave et al., 2022), which helps generate community buy-in and participation, and helps secure the longevity of a PLL.

Playful Learning Landscapes (PLL) draws on CBPR principles by eliciting community values, practices, and funds of knowledge and marrying these with principles from developmental science around high-quality learning environments. Activities for PLL are informed by community members through focus-group meetings, surveys, or interviews. These methods provide rich data about community values and practices, such as the importance of family unity or intergenerational learning, that can be intentionally incorporated into PLL designs. In addition, co-design activities, such as having design partners engage in prototype creation with arts and crafts (e.g., *bags of stuff*), can be used to elicit community members’ design ideas (Fails et al., 2013; Yip et al., 2013). This also allows researchers to capitalize on community assets and community funds of knowledge to ensure that PLL activities connect with and embody existing community cultural practices (Moll et al., 1992). Our approach thus starts by meeting community members where they are, hearing their concerns, and valuing their input. This part of the model allows our scientific research to be more adaptive to community interests and values.

### How children learn

The second part of the equation is brought to the table by the scientific community. Consensus exists in the relatively new *science of learning* (Meltzoff et al., 2009) that children learn best when the learning environment is active, engaging, meaningful, socially interactive, iterative, and joyful (Zosh et al., 2013; Hirsh-Pasek et al., 2015; Weisberg et al., 2016). Foundational to our reinvention of a new type of public square is the curation of activities that embody these six pillars, which together are found in what we call *playful learning*. That is, as part of the design process, we ask whether the PLL will include opportunities for active learning, engagement, meaningful learning, social interaction, iterative learning, and joy.

Playful learning has emerged as a term that describes a continuum, anchored on one end by free play and on the other by direct instruction, which is not play at all, with a range of play types in between, including guided play and games (Zosh et al., 2018; Hassinger-Das et al., 2020). Free play is a child-led and child-initiated experience that generally lacks an intentional learning objective. Direct instruction, on the other hand, has a clear learning objective, but is adult-led and adult-directed, often removing many of the key pillars from the experience (e.g., that it should be active and joyful). Guided play is situated between free play and direct instruction. It capitalizes on the learning pillars by providing children space to direct their learning while borrowing the best of direct instruction by incorporating a target learning goal. That goal can be scaffolded either by gentle guidance and questioning from an adult or through a well-planned and curated environment that supports children’s playful exploration, as in PLLs (Zosh et al., 2018).

Compared with direct instruction and free play, guided play holds a privileged spot in the literature in that it often produces strong learning outcomes while capitalizing on children’s organic method of engagement with their environment (Hirsh-Pasek et al., 2020; Hirsh-Pasek et al., 2022; Skene et al., 2022). For example, preschool-aged children taught about the properties of different shapes in a guided play condition learned more about the shapes compared with children taught similar information in direct instruction or free play contexts (Fisher et al., 2013). Play-based interventions also reveal the effectiveness of using guided play practices to promote learning. For example, children from under-resourced homes introduced to math concepts using play-based interventions (e.g., games) not only improved but also maintained skills learned through play in the weeks following the intervention (Siegler and Ramani, 2008; Scalise et al., 2018, 2020). A vocabulary play-based intervention for low-income preschoolers was also effective at promoting vocabulary growth when learning was scaffolded by an adult through guided play (Toub et al., 2018). In work with parents, preschool-aged children engaged in more math talk when parents were instructed to supplement a playful game with guided math-based talk compared with parents who did not engage in math-related talk during the play activity (Zippert et al., 2019). When teaching preschoolers fractions, parents who taught using guided play practices reported just as much math talk and more joy compared with those who taught in ways reflecting direct instruction approaches (Eason and Ramani, 2018). These studies highlight how guided play not only promotes learning but also joyful exchanges that promote high-quality interactions between children and their caregivers.

Environments designed to elicit guided play have also been shown to promote children’s social and cognitive development. Montessori and Tools of the Mind are two educational approaches that emphasize guided play practices in their curriculum (Montessori, 1964; Bodrova, 1997; Bodrova and Leong, 2007). Both approaches foster child agency in semi-structured playful learning environments where learning is scaffolded by the environment, as in the Montessori model, or by an adult. Preschoolers learning through these approaches show better outcomes on standardized assessments, better EF skills, and more advanced social skills compared with preschoolers in programs that adopt more traditional direct instruction curriculum (Lillard and Else-Quest, 2006; Blair and Raver, 2014; Diamond et al., 2019). These findings highlight that environments, in addition to adult–child interactions, can be intentionally designed to promote the guided play.

### What children learn

Finally, the question of what the learning goal should be is central to the design of an installation in PLL. Although children can master content through guided play, educational opportunities that build skills, such as collaboration, communication, content, critical thinking, creative innovation, and confidence (the 6 Cs) are as important as mastering content alone (Darling-Hammond et al., 2020; Hirsh-Pasek et al., 2020). In fact, there is considerable overlap between 6 C growth and school readiness outcomes. For example, the Early Development Index (EDI), an assessment used to evaluate kindergarten readiness for students residing in Orange County, CA, emphasizes readiness in the domains of language and cognitive development (such as content and communication), communication skills and general knowledge (such as collaboration, critical thinking, and creative innovation), physical wellbeing and motor development, and social and emotional development (such as communication, collaboration, and content). Fostering 6 C growth maps onto the same developmental outcomes that are important for educational success. Thus, environments that foster the 6 Cs set children on a trajectory for academic success and establish the habits of mind that promote lifelong learning.

## Applying the model

### Engage

Integrating these learning frameworks with community funds of knowledge is a complex process that requires strategic planning and strong partnerships. Implementation of our three-part equation begins by developing RPPs (Penuel and Gallagher, 2017; Farrell et al., 2021) with local community organizations that serve a target population of interest. We engage with community members to identify spaces where they want to see PLLs installed and then co-design activities for the space that reflect the community’s priorities, values, and cultural identity. By including members of the community in the design process, PLLs establish connections with the community’s cultural practices while elevating community voices and ownership.

For example, our first case study (Case Study 1) applied our model to a new cultural setting in Santa Ana, California. We aimed to deepen community engagement by involving parents and community members in every aspect of our design process. We were connected with parents from the Santa Ana community through an established RPP with trusted community leaders from the Santa Ana Early Learning Initiative (SAELI), a community-led partnership connecting caregivers with non-profit organizations. We worked closely with the founder of SAELI and the existing director, who also envisioned ways in which, as a community, multiple stakeholders could come together to support early learning outcomes for children 0–9 years of age. The importance of connecting with the directors of SAELI cannot be understated. They brought expertise and knowledge about the community and valuable insights about working with local families. They participated in planning meetings and invited us to attend the organization’s meetings, from which we learned about their 3-step design framework (namely, Discover and Dream, Design and Destiny, and Sustainability Plan), which we adopted and aligned with Playful Learning Principles in subsequent design sessions. Finally, SAELI leaders connected us with the parents who participated and engaged in our design sessions and continuously supported the process even during the COVID-19 pandemic.

Our second case study (Case Study 2) applied our model to early education settings in the greater Philadelphia area. An RPP was established with a local early childhood education (ECE) network that serves a diverse set of communities. Six early learning centers, serving approximately 50–100 families each, expressed interest in partnering with us. The centers vary by neighborhood and the communities served, which are diverse across religious affiliation, race and ethnicity, and socioeconomic status of students and families. For example, one of the centers focuses on serving predominantly Jewish communities and is located within a synagogue. Another center is located within and affiliated with a local church. Yet, another center is located within the center of the city and predominantly serves children living in downtown Philadelphia. The three remaining centers are located in the surrounding suburbs.

### Plan

Once an RPP is established, decisions about the project are co-planned to determine the project timeline, community-engagement activities, and each stakeholder’s role in the initiative. Communication about the community’s role in the design process ensures transparency and alignment with project goals. For example, community involvement as users, testers, informants, or design partners should be identified and a plan for situating communities in that role established early in the process (Chew et al., 2021).

With the Case Study 1, although our central focus was on co-designing with children and families, other vital stakeholders participated in our design process. For example, the Administrative Service Manager for the City of Santa Ana attended community project meetings, provided feedback on installation prototypes, and highlighted alignment between the current project and existing city projects. Government participation in our project led to city officials indicating their interest in implementing designs co-created with SAELI families into several upcoming park renovation projects. City funding of these projects is allowing us to install PLL in spaces throughout Santa Ana that were previously outside the scope of our research team’s budget. Integrating PLL into the city’s renovation process further increases the sustainability of the PLL model.

Similarly, although Case Study 2 designs will be installed in classrooms, our team worked closely with 30–40 stakeholders affiliated with our ECE partners, such as key personnel from the program’s education and curriculum teams, center directors, health and safety directors, and client relations specialists.

### Design

There are several avenues for the co-creation of playful learning spaces, depending on the role(s) community partners assume, which can differ based on funding, project timeline, and project goals. Co-design is an iterative process that capitalizes on community assets and builds on community expertise to optimize children’s learning experiences (Bonsignore et al., 2013). Both case studies highlight an approach that involves deep community involvement and illustrate the flexibility of the PLL development process to suit the needs and priorities of partners ranging from city officials to private organizations and to the children and families who subsequently use the installations.

**FIGURE 2:**
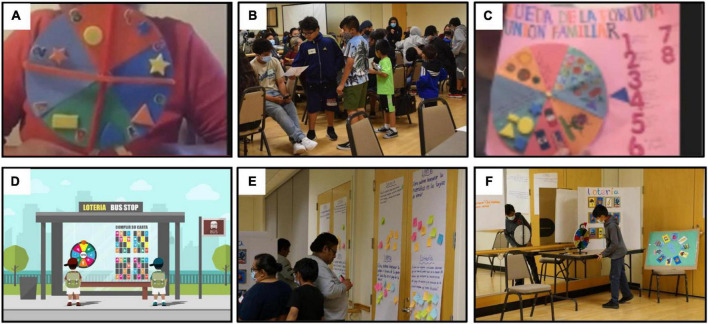
Co-design process for creating Playful Learning Landscapes with community members. Pictured here are six iterations of what became the Lotería game bus stop. **(A)** While brainstorming ideas, parents virtually shared their creative prototypes of different playful experiences with the group. **(B)** Parents play-tested their initial prototypes with their children and created new prototypes incorporating children’s ideas. **(C)** Next, parents shared their experience playing with their children and introduced their new design in a codesign breakout group, providing opportunities for other parents to give their feedback and collectively create more detailed prototypes and game ideas. **(D)** After much feedback from parents and children, our research team met to elaborate on parents’ values and designs, and aligned them to learning principles and early STEM concepts, creating refined prototypes of the PLLs. **(E)** To get feedback on the refined prototypes we asked parents to respond to prompts regarding how we could make the installation culturally relevant to Santa Ana and whether they thought the installations were engaging and meaningful. **(F)** Finally, children and families tested a series of physical prototypes of different PLLs.

Co-design in Case Study 1 included a series of seven virtual design sessions, led by Spanish-speaking facilitators, where Latine caregivers (*n* = 36) shared their family and local community experiences. One goal of these workshops was to identify locations for PLLs. Caregivers shared pictures of local spaces and told stories about daily activities they do with their children around the city. As one example, parents expressed their desire to redesign a park in the neighborhood to look more like the plazas of Mexico to pay homage to their cultural heritage. The park became a significant location central to our design activities and planning. Another goal of these workshops was to elicit cultural values and practices and co-design activities to fill these public spaces. Through conversations and structured activities, a core set of cultural values and practices important to families emerged, including intergenerational learning, heritage, and community engagement. With these values in mind, parents created and shared creative prototypes of different installation ideas with the broader group (see Figure 2, Panel A). This provided opportunities for parents to collectively create more detailed prototypes and playful game ideas. Our research team met to elaborate on parents’ values and designs, assuring that they aligned with learning principles and early STEM concepts, and creating refined prototypes of the PLLs. For example, Lotería bus stop emerged from a mother’s spinning wheel design for learning math concepts, which was subsequently refined to model a popular cultural game, Lotería, which incorporates science and math content (see Figure 2, Panel D). This design thus leveraged families’ collaboration, intergenerational learning, and heritage values through a familiar and enjoyable practice to promote learning through children’s observation, prediction, comparison of quantities, and problem solving.

**FIGURE 3:**
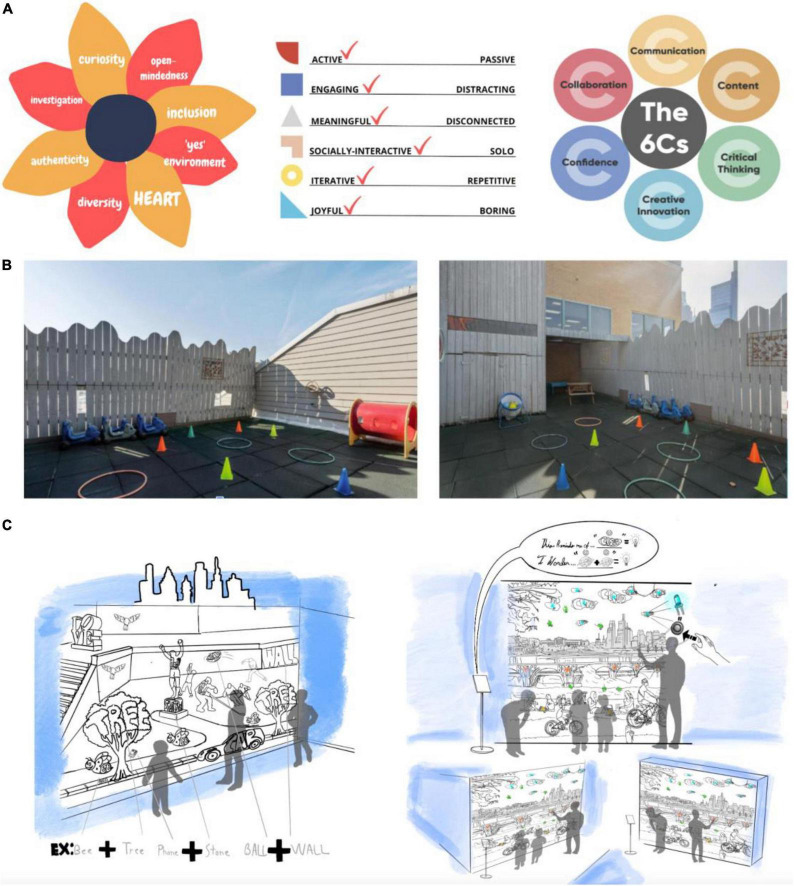
Pictured here are images and illustrations representing stages of the co-design process. A represents the guiding principles of the co-design process: **(A)** pinwheel representing the overarching community values identified by participants in the first workshop, a checklist of the pillars of play, and a graphic of the 6 C’s. **(B)** shows community-sourced images from one of the centers of their rooftop playground. Teachers expressed that they used to be able to see the city skyline from their playground but can no longer see it during the second workshop. **(C)** shows the designs created by a design team to reimagine what the playground pictured could look like with a playful learning installation. During the third workshop, teachers, directors, and other stakeholders provided feedback on the designs and discussed desired changes and adjustments.

Co-design in Case Study 2 involved three virtual design sessions. Workshops included participants (*n* = 35) from each of the six participating early learning centers. Community values were elicited in our first design workshop using a “Mad Libs” activity in which participants generated values by brainstorming learning- or principle-related adjectives that were important to them and coalesced around core themes they wanted the installations to invoke. Across the six centers, the core values that emerged from the workshops included diversity, curiosity, inclusion, and an “environment of yes” in which students are encouraged to explore their surroundings (see Figure 3). Teachers and center directors shared photographs from their centers of spaces important to them that they wanted to reimagine for their students. In addition, center-specific characters and images were sourced for the designs to capture their unique histories and traditions for “neighborhood flair.” For example, the center located within a synagogue selected images related to their cultural heritage, such as Noah’s Arc, Moses, and Hebrew letters and words. A suburban center selected images including flags reflecting the diverse international heritages of their teachers and hobbies teachers to partake in like camping and traveling. Teachers and curriculum specialists also provided expert opinions on the learning needs of their students. Science, technology, engineering, and mathematics (STEM) learning goals included: (1) Describe patterns, recognize shapes and numbers, and develop spatial language; (2) Practice investigation and observation skills; and (3) Solve problems using multiple methods. Learning goals for literacy included: (1) Recognize letters and sounds in the alphabet, identify words, and rhyming; (2) Allow children to express themselves through telling stories and dramatic play; and (3) Be curious, observe and describe people, places, and things around them. Finally, design considerations important to the community included: universal designs for learning to ensure students of all ages and ability levels can engage with the installations, flexibility of installment (so they can be relocated as desired), and incorporation of physical motion to allow children to take movement breaks throughout the day.

### Approve

Graphic designers and architects then drafted blueprints of the installation(s) based on community input solicited during co-design workshops. Blueprints were subsequently shared with the community for further input and discussion. This feedback loop continued until the final designs were approved by both research and community partners. This process ensured that each installation reflected the community’s values and embedded the principles of *how* and *what* of learning (ensuring that all three criteria of our science of learning model’s equation were met). Final designs also adhered to a set of considerations consistent with city regulations and best practices (e.g., no loose parts, minimal text, and inviting and engaging design).

In Case Study 1, we undertook an iterative process involving the research team and community members to draft blueprints of PLL installation prototypes. First, our research team conducted an inductive thematic qualitative analysis of co-design meeting transcripts to capture and understand the values and practices of Latine families at home and in their community spaces. Next, the research team (*n* = 11) met several times to establish a shared understanding of families’ stated values and experiences and review parents’ design ideas and prototypes. The research team then selected a subset of parents’ ideas and designs to elaborate on and ensured alignment with the science of learning and early STEM learning goals while maintaining parents’ values and experiences. Finally, two in-person co-design sessions were conducted with parents (*n* = 29) and children (*n* = 54) where families play-tested life-size mock-ups of PLL designs (see Figure 2, Panel F). To obtain feedback on these refined prototypes, caregivers responded to written and verbal prompts regarding how to make the activities more culturally relevant and how to improve families’ experiences using the installations. This iterative process continued until designs were approved and at all points maintained families’ values.

An iterative process was also used to create PLL prototypes in Case Study 2. Again here, our research team met several times to process themes from the co-design workshops to create a shared understanding of participants’ values and review design ideas and learning goals. Ideas for designs were aligned with the science of learning while still capturing the themes expressed by teachers and center directors during the design workshops. We developed a set of tailored installations designed to focus either on literacy and storytelling or science, technology, engineering, and math (STEM). For example (see Figure 3), several teachers from a center located in downtown Philadelphia expressed their desire to see the city skyline, which was once visible from their rooftop playground but is now eclipsed by a newly-constructed apartment building. At a subsequent workshop, teachers expressed their students’ need for opportunities to build phonetic skills including rhyming, sentence structure, and describing characters and settings in narratives. By the third workshop, in collaboration with designers, we designed an interactive skyline rooftop mural to fit their playground, embedded with rhyming words and images, and supplemented with verbal prompts to describe the city (see Panel C, Figure 3).

### Build

We began the process of fabrication and installation once the designs were approved by all key stakeholders. In both case studies, local fabricators and architects were prioritized as yet another way to include businesses from the target community in the creation of these PLLs. Where appropriate, community members were invited to help with the fabrication and installation process (e.g., Urban Thinkscape; Hassinger-Das et al., 2020).

In summary, these two case studies illustrate how our model can be refined and applied to new cultural contexts (e.g., a largely Hispanic/Latine community in CA) and settings (e.g., early learning centers). Even during the COVID-19 pandemic, which placed immense stress on communities and educators, families and teachers were meaningfully engaged in our design workshops. Their sustained engagement speaks volumes to what happens when scientists involve community members in the scientific process.

## Evaluating the model

Another strength of our model is that it can be rigorously and reliably evaluated. For example, scientists can design observational and methodological protocols to examine the impact of a PLL on the surrounding community. This part of our process also incorporates the principles of CBPR. Indeed, in Philadelphia, members of the community became part of the scientific team to research the impact of the installations on caregiver–child behavior and child outcomes (Hassinger-Das et al., 2020, 2021). Evaluation of PLLs in previous work reveals that PLL outcomes are measurable and align with our goals of increasing adult–child interaction quality and language use, building positive attitudes and beliefs about playful learning, encouraging ownership of local spaces, and increasing civic engagement (Ridge et al., 2015; Hanner et al., 2019; Bustamante et al., 2020; Hassinger-Das et al., 2020; Gaudreau et al., 2021; Shivaram et al., 2021).

Observations of caregiver–child and teacher–child interactions in our two case studies described in this article will provide additional evidence about the impact PLLs have on interactions and child outcomes. Importantly, in both projects, we are actively taking steps to ensure items in our assessments are culturally appropriate and reflect diverse cultural contexts. For example, the observational protocols being developed to evaluate Case Study 1 are being translated and adapted with community input to reflect the ways in which caregivers express different constructs (e.g., numeric and spatial skills) both behaviorally and linguistically (Melzi et al., 2022). This ensures that our observations capture culture-specific behavior and language relevant to our outcomes of interest.

Previous work has also evaluated caregiver views on play and learning after interacting with PLL. Caregivers with more exposure to PLLs indicated a greater understanding of the connection between play and learning (Grob et al., 2017). A similar approach will be implemented to evaluate play and learning attitudes among Spanish-speaking caregivers who reside in the Case Study 1 community. Surveys will be adapted to specific cultural and community contexts, for example, by engaging in an iterative translation process and soliciting feedback from community members about the relevance of survey items to their culture and community. This process allows us to generate methodological tools that are appropriate to the local community context before administering them in the field.

**FIGURE 4:**
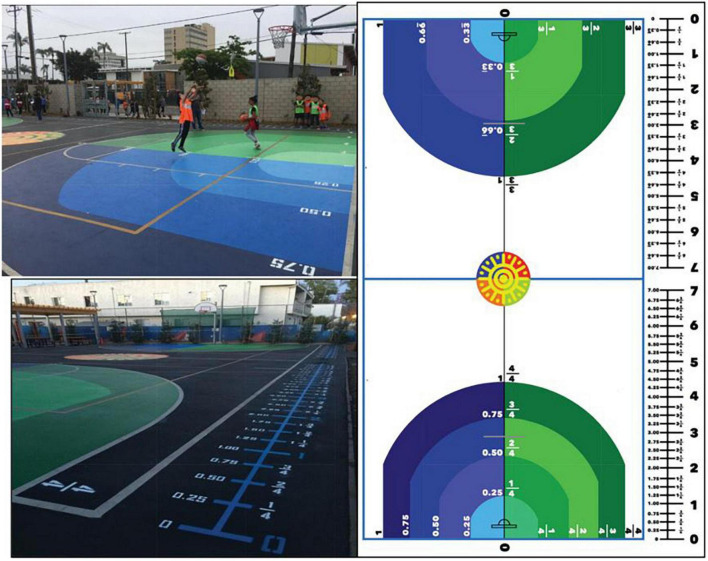
Fraction Ball installation in Santa Ana, CA from Bustamante et al. (2022).

Child learning outcomes are another measurable outcome of PLL. Recently published work investigated the impact that a PLL called Fraction Ball had on students’ fraction and decimal number learning (Bustamante et al., 2022). Fraction Ball is a reimagined basketball court, where lines are added to the court indicating fractions and decimals as a function of distance away from the basket (see Figure 4). After modifying courts at an elementary school in Santa Ana, California, fifth and sixth grade students were randomly assigned to play Fraction Ball during their physical education (PE) class period or to engage in PE as usual. Students who played Fraction Ball showed greater improvement in fraction and decimal number understanding from pretest to posttest than students who did not play Fraction Ball and continued business as usual (Bustamante et al., 2022). Drawing from this approach, we plan to examine the impact that PLLs installed in diverse community spaces (Case Study 1) and early learning settings (Case Study 2) have on children’s learning. Our team will use data from the Early Development Index (EDI), a teacher-reported population-based assessment of school readiness that evaluates child development in physical health, social competence, emotional maturity, language and cognitive development, and communication to examine whether population-level changes in school readiness emerge in the years following PLL installation in our Case Study 1 community. This will have important implications for urban design and city planning in the long term.

Finally, the impact of PLL on community wellbeing can be evaluated. During our co-design process, community members were given opportunities to meet others who live in their community and to engage with stakeholders, such as funders, policymakers, and researchers. We expect this process to foster civic engagement and increase social cohesion, which can strengthen economic health and wellbeing, particularly in underserved neighborhoods (Hadani et al., 2021). In collaboration with our policy partners at the Brookings Institution, a new metrics framework provides policy-relevant recommendations for measuring these types of community-level outcomes (Hadani et al., 2021). Specifically, data on the frequency of visits to a PLL site, length of visit, community engagement in volunteer activities at the site, small business impact, and accessibility of the site for people with special needs will be acquired to provide additional documentation about how PLLs impact communities. This information will be leveraged to inform and scale our PLL work in future.

## Discussion

In this paper, we offer a new model a new model for conducting developmental and educational research that integrates community input and values with the science of *how* and *what* children learn. This is an evidence-based approach that deviates from traditional developmental science research by actively including communities in research design and implementation from the outset. Implementation and evaluation of this model suggest that it not only changes human behavior in public and educational spaces but can do so in culturally sensitive and inclusive ways that are also supportive of high-quality adult–child interactions and child outcomes. In each of our case studies, RPPs were established with trusted community sources, which allowed our research team to develop relationships with local community members, such as parents and educators. This was essential to ensure participation in design sessions, where community input on important values, practices, and design ideas was solicited. Community funds of knowledge were then aligned with evidence-based research from the science of learning about how children learn best and what skills support strong learning outcomes through an iterative process. This yielded installations that reflected the priorities of both the community and scientific practice.

In addition to successfully implementing the model, we showed how this method can be empirically validated in culturally responsive ways. This involves not only designing with communities but also turning to communities as a source of information regarding what is culturally relevant and appropriate to measure. Observational protocols and other methodological tools, developed with community input, have been used to evaluate the impact of PLL on families, children, and communities in past PLL projects. We anticipate these measures will reveal the impact that PLLs designed in Case Studies 1 and 2 have on child-learning outcomes, as well as community-wide attitudes and beliefs in the value of playful learning.

This model for conducting psychological research responds to calls from the scientific community to engage in translational research and advance scholarship that fosters a more diverse equitable and inclusive science (Gruber et al., 2019; Rodriguez Espinosa and Verney, 2020; Buchanan et al., 2021; Haden et al., 2022). Translational research offers opportunities not only to apply laboratory-based findings to real-world applications but also to learn about development in real-world contexts (Golinkoff et al., 2017; Thompson, 2019). By inviting community members, including families, government officials, and educators, to take part in our scientific endeavors as research partners and design collaborators, we create space to learn and advance our science together. Communities are afforded opportunities to learn about developmental research from developmental and educational researchers, which opens lines of communication to translate science to families and policymakers. Likewise, researchers have opportunities to garner rich information about family values and dynamics, educational practices, and child development directly from communities in non-experimental settings and with non-traditional methodological approaches. For example, our co-design process in Santa Ana not only aided in the development of designs that reflected the community’s cultural values but also it provided our research team with descriptive data about family practices and assets that Latine families use to engage children in STEM learning (Bermudez et al., under review)^1^. In addition, by including families as data ambassadors in PLL projects, we further invite collaboration from diverse populations to inform research questions and design. This allows for a more inclusive science that can better engage with and reflect diverse populations by engaging them in new ways throughout the research process. Indeed, this type of community-based science asks researchers to consider whose voices are being centered in an initiative and develop scientific practices and methodologies that reflect this. The PLL initiative aims to respond to these calls by integrating community-based participatory research principles with scientific rigor.

### Limitations

Though the PLL model is a promising addition to the developmental and educational psychology literature, the Case Studies described in this manuscript are part of an initiative that is still relatively new. Additional implementation and research of the model are needed across a wider range of community contexts, such as additional non-dominant cultural communities and in a wider range of school types, to determine its effectiveness, identify aspects of our model that could use refinement or improvement, and foster opportunities to build connections with more community stakeholders. Indeed, past research has found that community involvement using a culturally responsive approach to co-design engenders increased the sense of ownership of PLLs (Bustamante et al., 2019; Belgrave et al., 2022). Developing a sense of ownership surrounding PLLs can lead to increased use of local spaces, which in turn increases the sustainability of PLL installations long-term and creates models for future PLL installations in other communities looking to adopt this approach. Existing research thus points to the effectiveness of our approach to uphold and reflect community values. However, more work is needed to definitively establish direct PLL impacts on child learning outcomes and overall community wellbeing and civic engagement.

Furthermore, the scope of impact of this work will be made clearer with ongoing efforts to scale PLL through policy innovations and partnerships. While PLL has been successfully implemented in several projects over the past few years (e.g., as described by Bustamante et al., 2020; Hassinger-Das et al., 2020, and in this manuscript), the process for scaling this initiative is in progress. There is evidence supportive of the scalability of both community-based RPP research and playful learning innovations. Research conducted in New Hampshire (Hirsh-Pasek et al., 2022; Nesbitt et al., under review^2^) suggests that the PLL model enacted through facilitated conversations with educators and school leadership can be implemented at the school district level and among populations of older children. In addition, international efforts to implement playful learning models, such as those in Finland, Singapore, India, Canada, and China (Gibbs et al., 2022) point to the widespread adoption of the principles described here that can have global significance and be applied across a wide range of cultural contexts. Coupling the community-based RPP approach with playful learning innovations at scale has the potential to fundamentally reshape how we approach educational priorities and urban development. The generalizability of our model to new cultures and contexts and the integration of this initiative across different sectors (e.g., public policy, education, and government) will advance our understanding of how to successfully conduct translational research that is inclusive and equitable.

## Conclusion

With a foundation built upon research with consensus from the scientific community, we further show that scientific theory and application can be built upon areas of agreement, rather than gaps. That is, scientists often conduct research that aims to provide answers to missing questions or to illuminate what we do not yet know about a particular psychological phenomenon. While this is an important lever for advancing scientific inquiry, we offer a complementary approach that asks not only what we do not know but also what we do know and how we can build on past successes for a more successful future. Areas in which relative consensus exists can be used to inform practical applications of research and methods for scaling those applications to the broader community level. PLL is one such example, taking what we know from the science of learning and implementing it into practice to impact how adults and children interact and learn in everyday spaces.

The PLL initiative sits at the nexus of the built and social environments. The unique process of community co-design integrates physical settings with learning goals that align with social customs, practices, and values. By bringing research from laboratories to community spaces, the science of learning can be infused into the everyday places of children’s lives. The PLL model thereby offers an opportunity to bridge gaps between research and practice in a human-centered culturally relevant way. As the initiative expands, we will be able to evaluate in greater detail how different moderators (e.g., demographic composition of communities) impact various outcomes (e.g., learning gains and changes in community beliefs). Applying the model described in this manuscript to the development of playful learning cities nationally and internationally has the potential to yield a future generation of leaders with the skills and dispositions needed for global cooperation and success.

## Ethics statement

The studies involving human participants were reviewed and approved by Human Research Protections (HRP), University of California, Irvine, Irvine, CA and Institutional Review Board (IRB), Temple University, Philadelphia, PA. Written informed consent to participate in this study was provided by the participants’ legal guardian/next of kin. Written informed consent was obtained from the individual(s), and minor(s)’ legal guardian/next of kin, for the publication of any potentially identifiable images or data included in this article.

## Author contributions

AP, KO, VB, JS, JA, AB, and KH-P contributed to case study 1. AP, KF, RT, HG, and KH-P contributed to case study 2. AP wrote the first draft of the manuscript. KO, KF, VB, RT, and JS wrote sections of the manuscript. HG, JA, AB, and KH-P provided critical revisions to the manuscript. All authors contributed to the case study design and implementation and contributed to manuscript revision, read, and approved the submitted version.

## Footnotes

1 Bermudez, V. N., Salazar, J., Garcia, L., Ochoa, K. D., Pesch, A., Roldan, W., et al. (under review). *Designing culturally situated playful environments for early STEM learning in a Latine Community*.

2 Nesbitt, K. T., Blinkoff, E., Gunersel, A. B., and Hirsh-Pasek, K. (under review). Play-based learning in New Hampshire kindergarten classrooms.
